# ^131^I–Tositumomab in lymphoma

**DOI:** 10.3747/co.v16i5.385

**Published:** 2009-09

**Authors:** M.C. Cheung, J.A. MacEachern, A.E. Haynes, R.M. Meyer, K. Imrie, the Members of the Hematology Disease Site Group of Cancer Care Ontario’s Program in Evidence-Based Care

**Affiliations:** * Cancer Care Ontario, Program in Evidence-Based Care, McMaster University, Hamilton, ON; † Division of Hematology/Medical Oncology, Odette Cancer Centre, Sunnybrook Health Sciences Centre, University of Toronto, Toronto, ON; ‡ Division of Oncology, Grand River Regional Cancer Centre, Kitchener, ON; § Department of Clinical Epidemiology and Biostatistics, McMaster University, Hamilton, ON; || Divisions of Oncology, Medicine, and Community Health and Epidemiology, Queen’s University, Kingston, ON

**Keywords:** ^131^I–Tositumomab, Bexxar, indolent lymphoma, systematic review

## Abstract

Radioimmunoconjugates are radioisotope-bound monoclonal antibodies that target radiation specifically to sites of lymphoma involvement. Initial studies of ^131^I–tositumomab in non-Hodgkin lymphoma (nhl) have suggested benefit in patients with relapsed or refractory indolent disease. However, the routine adoption of this agent is tempered by concerns about associated toxicities and unclear long-term benefit. Based on a comprehensive search for studies on ^131^I–tositumomab use in lymphoma, this systematic review summarizes and evaluates the evidence on

- the benefits and risks of this novel therapy,
- the predictors for response and toxicity, and
- the role of dosimetry and imaging studies before treatment.

We identified 18 trials investigating the use of ^131^I–tositumomab for the treatment of adult patients with nhl. In trials of patients with relapsed or refractory indolent nhl, overall response rates ranged from 67% to 83%. In patients with follicular nhl refractory to the monoclonal antibody rituximab, response rates remained high (65%–72%). However, in rituximab-naïve patients with relapsed or refractory indolent or transformed nhl, improvements in time to progression or survival have not been clearly established. ^131^I–Tositumomab is an active agent in relapsed and refractory non-Hodgkin lymphoma that should be considered in selected patients.

## 1. INTRODUCTION

Non-Hodgkin lymphomas (nhls) constitute a heterogeneous group of malignancies with variable presentations that range from indolent to aggressive 1. Patients with follicular and other indolent lymphomas can sustain prolonged remission periods, but they eventually relapse and require subsequent courses of therapy that lead to fewer and shorter remissions 2. Novel treatment options are necessary to improve the natural history of this condition. Rituximab is a chimeric monoclonal antibody directed against the CD20 surface antigen found on most B-cell lymphomas 3. Although rituximab represents an important advance in indolent disease because of its efficacy, short duration of therapy, and acceptable toxicity profile 4, relapse remains inevitable. Therapies that are more effective are thus needed for patients who are refractory to or who relapse after currently available treatments, including rituximab.

Radioimmunoconjugates are monoclonal antibodies bound to radioisotopes, and this emerging class of agents has activity in lymphoma. These agents allow for the delivery of targeted radiation therapy with the binding of the monoclonal antibody to antigens on the surface of malignant cells. ^131^I–Tositumomab (Bexxar: Corixa Corporation, South San Francisco, CA, and GlaxoSmithKline, Philadelphia, PA, U.S.A.) is a radioimmunoconjugate consisting of an anti-CD20 murine monoclonal antibody (tositumomab) covalently bound to the gamma-emitting radioactive isotope ^131^I 5,6. Initial studies have reported on the use of ^131^I–tositumomab in patients with refractory or relapsed low-grade, follicular, or transformed lymphoma. Further research is exploring the role of this compound in other settings, including in patients with aggressive-histology lymphomas and in the setting of stem-cell transplantation. However, the routine adoption of this agent is tempered by concerns about increased costs, complex dosimetry requirements, and possible toxicities. With the recent availability of radioimmunoconjugates, a careful review of the risks and benefits of such therapy is warranted. The aim of the present systematic review is to address the following questions in patients with lymphoma of any type or stage:

- What are the benefits associated with treatment with ^131^I–tositumomab?
- What are the toxicities associated with the use of ^131^I–tositumomab?
- Which patients are more or less likely to benefit from treatment with ^131^I–tositumomab?
- Is imaging or dosimetry required for therapy to be safe and effective?

## 2. MATERIALS AND METHODS

The methodology guiding this systematic review was developed by the Cancer Care Ontario (cco) Program in Evidence-Based Care (pebc) according to the practice guidelines development cycle 7. Members of the pebc Hematology Disease Site Group (dsg) selected, reviewed, and interpreted the evidence. The Hematology dsg has 25 members, including hematologists, medical and radiation oncologists, an epidemiologist, and two lay representatives.

### 2.1 Literature Search

We searched the medline (1966 to July 2005), embase (1980 to July 2005), and Cochrane Library (2005, Issue 3) databases using the search strategy shown in Table I. In addition, we searched the conference proceedings of the American Society of Hematology (ash) for 2000 to 2004 and those of the American Society of Clinical Oncology (asco) for 2000 to 2005. Reference lists of relevant trials and reviews were searched for additional publications. In addition, the authors searched their personal files. The Canadian Medical Association Infobase (mdm.ca/cpgsnew/cpgs/index.asp), the National Guidelines Clearinghouse (www.guideline.gov/index.asp), and the U.K. National Institute for Health and Clinical Excellence (www.nice.org.uk/) were also searched for existing evidence-based practice guidelines.

### 2.2 Inclusion Criteria

Randomized controlled trials, other comparative trials, prospective single-arm trials, systematic reviews (with or without meta-analyses), and evidence-based practice guidelines were considered for this review of the evidence if they met the following criteria:

- Study of adult patients with lymphoma of any type, at any stage, and for any performance status
- ^131^I–Tositumomab studied as a single agent or in combination with other regimens
- Results reported for one or more of the following outcomes: survival, quality of life (qol), time to progression (ttp), response duration, response rate, adverse effects, tumour dosimetry or imaging
- Report published in English

Letters, comments, and editorial publications were excluded. Conference abstracts that preceded full-paper final results were not included; however, abstracts that provided updated results or novel data were included for further data abstraction.

### 2.3 Data Extraction and Interpretative Summary

Relevant articles and abstracts were selected in an unblinded manner independently by two members of the Hematology dsg. Data were extracted and summarized to address the following questions regarding adult patients with lymphoma of any type, at any stage, and for any performance status:

- Is ^131^I–tositumomab effective in improving survival, qol, ttp, response duration, or response rate?
- What are the toxicities associated with the use of ^131^I–tositumomab?
- Which patients are more or less likely to benefit from treatment with ^131^I–tositumomab?
- Is imaging or dosimetry required for therapy to be safe and effective?

The analysis of the data uses descriptive statistics. Categorical variables are reported as numbers and proportions, and continuous data are reported as means and standard deviations. The heterogeneity among studies precluded any pooling of results using formal meta-analytic techniques.

## 3. RESULTS

We identified 255 citations in the literature searches of medline, embase, and the Cochrane Library, including twenty-one full publications of eleven trials. Eleven abstracts of seven trials were identified from the conference proceedings of ash and asco. One additional abstract was identified that provided qol data that were not reported in the relevant full publication. Only the most recent abstract or full publication was referenced for each trial, except where additional data were available in previous publications (Table II^a^).

In total, this systematic review includes eighteen trials investigating the use of ^131^I–tositumomab for the treatment of adult patients with nhl. No systematic reviews, meta-analyses, or evidence-based practice guidelines were identified. We divided the trials into two categories based on patient treatment history: previously untreated 25,27,29,31,35 and previously treated patients with nhl. The “previously treated” category was further divided into randomized 22 and single-arm trials of ^131^I–tositumomab. The single-arm trials included reports of patients with disease relapsed or refractory to chemotherapy without prior rituximab 5,8,11,12,19,28; disease relapsed or refractory to rituximab alone 34,39; disease treated with ^131^I–tositumomab conditioning for autologous stem-cell transplantation (asct) 17,40; and disease treated with ^131^I–tositumomab in alternative regimens or alternative populations of previously treated patients 23,32.

## 3.1 Patients with Previously Treated NHL

### 3.1.1 Study Quality

Only one of the thirteen trials of ^131^I–tositumomab in patients with previously treated nhl was a randomized controlled trial 22. That trial has been published in abstract form only, and therefore little information regarding study quality was reported. However, the authors did report that the trial was multicentred and open-label. The 78 study patients were randomized either to ^131^I–tositumomab or to unlabelled tositumomab and were followed for a median of 42.6 months. No sample-size calculation was provided.

One single-arm trial, reported as a full publication by Kaminski *et al.* 5, compared each patient’s duration of response after ^131^I–tositumomab with the duration of response to their last qualifying chemotherapy regimen (“paired control”). The remaining studies were single-arm noncomparative phase i or ii trials. Eight of those trials 5,8,11,12,17,19,22,28,34,40 have been fully published, with sample sizes ranging from 11 patients to 60 patients. The remaining three trials 23,32,39 have been published in abstract form only, with sample sizes ranging from 11 patients to 32 patients. Eight of twelve single-arm trials reported median follow-up times that ranged from 12 months 11 to 39 months 34.

### 3.1.2 Study Characteristics

Table III presents study and patient characteristics for the trials of ^131^I–tositumomab in patients with previously treated nhl. The randomized trial reported by Davis *et al.* 22 included patients with CD20+ nhl that was relapsed or refractory (defined as progression within 1 year of treatment) to a regimen containing either an anthracycline, an anthracenedione, or an alkylating agent. Patients were randomized to either ^131^I– tositumomab (*n* = 42) or to unlabelled tositumomab (*n* = 36). Patients who did not respond to unlabelled tositumomab could cross over to the ^131^I–tositumomab arm if they did not have a human anti-mouse antibody (hama) response. The authors did not report the doses given to patients in either arm. Patient characteristics were well matched between the two treatment arms.

Six of the single-arm trials enrolled patients with nhl that was relapsed or refractory to chemotherapy without rituximab: one phase i dose-escalation trial 8, one phase i/ii dose-escalation trial 12, and four phase ii trials 5,11,19,28. One of the phase ii trials used each patient as a self paired control for comparing the duration of response to ^131^I–tositumomab with the duration of response in the patient’s last qualifying chemotherapy regimen 5. The phase i trial reported by Press *et al.* 8 was a dose-escalation study that provided separate outcomes data for the 12 patients who received therapeutic doses of ^131^I–tositumomab. Gopal *et al.* 10 reported a study that compared patients in sequential trials reported by Press *et al.* 22,27 (treatment group, *n* = 27) with a historical control group of patients who received conventional high-dose therapy and asct (control group, *n* = 98).

Two single-arm phase ii trials enrolled patients with nhl relapsed or refractory to rituximab, with or without chemotherapy. The first trial, reported by Horning *et al.* 34 included patients who were relapsed or refractory to rituximab. Nair *et al.* 39 reported a trial that enrolled patients with CD20+ nhl refractory to chemotherapy and rituximab.

Two single-arm phase i dose escalation trials treated patients who had chemotherapy-resistant nhl with a regimen including ^131^I–tositumomab conditioning for asct 17,40.

Mones *et al.* 32 reported the results of a phase i trial that enrolled patients who had relapsed or refractory low-grade nhl and more than 25% bone marrow involvement. The first cohort of patients received ^131^I– tositumomab at a total body dose of 45 cGy, with incremental increases of 10 cGy for subsequent cohorts.

Four of the single-arm trials reported subgroup data for patients with transformed nhl 5,12,19,28. Three additional trials 17,23,34 reported that 12%–23% of enrolled patients had transformed nhl, but they did not provide outcomes data for that subgroup of patients. An integrated pooled analysis of five of these studies 5,12,19,28,34 reported outcomes unique to this subgroup of patients 43,44,45. In this population of 71 evaluable patients with transformed nhl, the median time from diagnosis to therapy was 74 months, and the median time from transformation was 21 months 45.

### 3.1.3 Response Rate

In previously treated patients, objective response rates ranged from 85% to 100%, with complete response (cr) rates of 20%–84% 5,8,11,12,17,19,22,23,28,32,34,39,40 (Table IV). In the randomized trial 22, a statistically significant difference was observed in objective response between the ^131^I–tositumomab group and the unlabelled tositumomab group (55% vs. 19%), with cr rates of 33% versus 8% (statistical significance not reported). Response rates in rituximab-naïve patients ranged from 57% 19 to 100% 8 with cr rates from 20% 5 to 83% 8. Response to ^131^I–tositumomab appeared to compare favourably with the response to the preceding line of therapy (chemotherapy alone) 5. For patients that had relapsed after or were refractory to rituximab (with or without chemotherapy), response rates were 65% 34 and 72% 39 respectively.

The response rates were 65% 40 and 87% 17 in the trials that treated patients with ^131^I–tositumomab as part of multi-agent chemotherapy conditioning for asct, with cr rates of 57% 40 and 77% 17.

One trial treated patients with more than 25% bone marrow involvement (a relative contraindication to the use of ^131^I–tositumomab) and observed an objective response rate of 18% in 11 patients 32.

A pooled analysis of five trials 43,44,45 provided response data for the subgroup of patients with transformed nhl. A pooled response rate of 39% with a cr rate of 25% was reported.

### 3.1.4 Time to Progression

In previously treated patients, ttp data were reported for ten trials (Table IV). One randomized trial 22 reported that median ttp was longer in the ^131^I–tositumomab arm as compared with the unlabelled tositumomab arm (6.3 months vs. 5.5 months, *p* = 0.031). Six single- arm trials of patients who were previously treated with chemotherapy or rituximab, or both 5,12,19,28,34, or with prior ^131^I–tositumomab 23, reported median ttp ranges from 8.4 months to 12 months. One trial reported a 1-year progression-free survival of 66% 11.

### 3.1.5 Response Duration

Data on response duration in previously treated patients were reported in eight trials (Table IV). In the randomized trial 22, median response duration was not reached in the ^131^I–tositumomab arm; it was 28.1 months in the unlabelled tositumomab arm (*p* = not reported). In previously treated patients who had not received rituximab, median response duration ranged from 6.5 months 5 to 15 months 6. In the trial that compared ^131^I–tositumomab response with that attained for last chemotherapy regimen in the same patients 5, 17 of 60 patients achieved a response duration after ^131^I–tositumomab that was equivalent to their most recent lymphoma treatment; 53% achieved a longer response duration after ^131^I–tositumomab (*p* < 0.001). In individuals with transformed nhl, the pooled analysis of five trials documented a median response duration of 20 months. In addition, of the 25% of individuals who attained a cr, median response duration reached 36.5 months 45.

### 3.1.6 Survival

In the trials that included patients who had nhl relapsed or refractory to chemotherapy without rituximab, the median os ranged from 21 months 8 to 41 months 12, with two trials reporting that the median os was not reached at 12 months 11 and 36 months 28 of follow-up (Table IV). In patients with disease that was relapsed or refractory to rituximab, median os had not yet been reached at 39 months 34. In patients who received ^131^I–tositumomab conditioning for asct, one trial reported a median os of 36 months 40, and another trial reported a 2-year os of 83% 17.

### 3.1.7 Quality of Life

Only one of the thirteen trials of ^131^I–tositumomab in previously treated patients reported data on qol 20. The European Organization for Research and Treatment of Cancer quality of life questionnaire was administered to the patient cohort receiving ^131^I–tositumomab after previous lymphoma treatment without rituximab. The authors reported that the scales for emotional function, social function, global health status, nausea/vomiting, and appetite loss demonstrated statistically significant improvements at one or more time points; however, no data or *p* values were reported.

### 3.1.8 Adverse Events

The randomized trial 22 comparing ^131^I–tositumomab with unlabelled tositumomab reported comparative grade 4 hematologic toxicities. Thrombocytopenia (12% vs. 0%), neutropenia (17% vs. 3%), and anemia (5% vs. 0%) occurred more frequently with radioimmunotherapy, although whether these differences were statistically significant was not reported (Table V).

The rates of adverse events were similar in patients who had 34,39 and who had not 5,8,11,12,19,28 received prior rituximab. Myelosuppression was common, but tended to be delayed in onset, with cytopenia nadirs occurring 7–9 weeks after treatment. Eight trials reported on the rate of infection, with grades 1–4 infections occurring in 21%–55% of patients 5,8,11,12,19,23,34. The rate of hospitalization from infection was reported in three trials and ranged from 2% to 15% 5,23,28. Non-hematologic toxicity was common (reported in 80% of patients), generally mild, and related to drug infusion. Grades 1 and 2 adverse events occurred in a high proportion of patients in all trials, with the most common events being headache, fever, chills, infection, nausea, and vomiting. Table V summarizes these adverse events.

The rate of hama response was reported in ten single-arm trials, occurring in 0%–35% of patients 5,8,11,12,19,17,23,28,34,40. Hypothyroidism was reported in six trials, and for the ^131^I–tositumomab arm of the randomized trial, it occurred in 7%–42% of patients 5,8,11,12,22,23,34.

The rate of myelodysplastic syndrome (mds) was reported in eight trials and ranged from 0% to 9% 5,8,9,11,12,23,28,34,40. One study that included patients from six trials and an expanded access program reported on mds and acute myeloid leukemia (aml) in patients treated with ^131^I–tositumomab 16: 35 of 1071 patients developed mds or aml for an annualized incidence of 1.4% per year (95% confidence interval: 1.0% to 2.0% per year).

### 3.1.9 Prognostic Factors

Predictors for overall response included tumour burden below 500 g 5, grade i or ii disease and tumour size 7 cm or less 34, lymph node diameter less than 5 cm 28, low-grade nhl 5,12, bone marrow involvement 5, fewer than 4 prior chemotherapy regimens 5, and no prior radiotherapy 5. The prior use of 2 or more chemotherapy regimens was associated with a shorter duration of remission 28.

### 3.1.10 Dosimetry and Imaging

Dosimetry is a method of estimating the dose of radiation administered to specific organs. Imaging refers to the evaluation of gamma images to ensure that drug biodistribution is appropriate 46. Dosimetry is required to determine the dose of ^131^I–tositumomab to be administered 21,47, and it was used in all trials in patients with previously treated nhl. No dose–response relationship was noted between absorbed dose and tumour response 21. Also, no correlation was observed between total body tumour burden and objective response or toxicity 21.

### 3.2 Patients with Previously Untreated NHL

#### 3.2.1 Study Quality

No randomized controlled trials of ^131^I–tositumomab in patients with previously untreated nhl were identified. All of the five studies located were single-arm noncomparative phase ii trials with sample sizes ranging from 13 patients to 90 patients 25,27,29,31,35. Median follow-up ranged from 11 months to 61.2 months.

#### 3.2.2 Study Characteristics

Table VI details the study and patient characteristics of trials of ^131^I–tositumomab in patients with previously untreated nhl.

#### 3.2.3 Response Rate

Table VII presents response data for the five trials of ^131^I–tositumomab in the previously untreated patient population. In the four trials of ^131^I–tositumomab alone 35 or after chemotherapy 25,29,31, objective response rates ranged from 90% to 100%, with cr rates from 67% to 83%. In a trial of sequential therapy with ^131^I–tositumomab followed by chop (cyclophosphamide, doxorubicin, vincristine, prednisone) chemotherapy (×6 cycles) in patients with mantle-cell nhl, the response rate was 83% after ^131^I–tositumomab (cr rate: 50%) and by intention-to-treat analysis, the response rate was 75% after chop (all of which were crs) 27.

#### 3.2.4 Time to Progression

Table VII presents data on ttp. One trial reported a median ttp of 73.2 months 35 and another reported a 2-year progression-free survival of 81% 25. Median ttp had not yet been reached in two other trials in which the median follow-up periods were 28 and 53 months 29,31.

#### 3.2.5 Response Duration

Only one trial 27 reported median response duration, which had not yet been reached after a median follow-up of 11 months (Table VII). Among the subset of patients who experienced a cr 35, 40 of 57 had experienced an ongoing cr for 4.3–7.7 years. A third trial 29 reported that the median duration of cr was not reached after a median follow-up of 52.8 months, with 72% of 29 patients with a cr remaining in remission.

#### 3.2.6 Survival

Two trials reported survival data for previously untreated patients (Table VII). One reported a 5-year os of 89% 35, and another reported a 2-year os of 97% 25.

#### 3.2.7 Quality of Life

None of the trials of patients with previously untreated nhl reported data on qol.

#### 3.2.8 Adverse Events

Table VIII summarizes adverse events in the previously untreated population. Grades 3 and 4 thrombocytopenia ranged from 11% to 29%; neutropenia, from 13% to 34%; and anemia, from 0% to 3% 25,29,31,35. Grade 3 infection occurred in 2% of patients 25, and febrile neutropenia was reported as 0% in one trial 35 and 42% in another 27. One trial reported a 0% rate of hospitalization as a result of infection 35; no other trials reported hospitalization data.

Elevated thyroid-stimulating hormone occurred in 7%–12% of patients 25,29,35, and hama occurred in 0%–63% 27,29,31,35, with mds or aml occurring in 0%–3% of patients 25,31,35.

#### 3.2.9 Prognostic Factors

One trial reported on predictive factors 35. Nodal diameters of 5 cm or more were associated with lower response rates, and bone marrow involvement was also associated with lower response rates. Only bone marrow involvement had a significant effect on progression-free survival, predicting for a worse outcome.

#### 3.2.10 Dosimetry and Imaging

All of the trials that enrolled patients with previously untreated nhl used dosimetry and imaging in the trial protocol. Dosimetry is part of the ^131^I–tositumomab regimen. One publication 38 provided updated data on patients in three other publications 36,37,47 and on an additional 19 patients. The authors reported that, for patients with previously untreated follicular nhl who received ^131^I–tositumomab, those with tumours receiving the highest radiation doses were more likely to achieve a cr; however, that association was not statistically significant.

## 4. DISCUSSION

The development of the monoclonal antibody rituximab has significantly advanced the treatment of lymphomas that express the target CD20 antigen. The anti-lymphoma benefit of rituximab is likely multifactorial, including antibody- and complement-dependent cellular cytotoxicity mechanisms 4. In addition to these immunobiologic effects, radioimmunoconjugates have the potential to direct radiation exclusively to the site of disease involvement, minimizing exposure to uninvolved organs. The adoption of these agents will depend on whether the incremental anti-lymphoma activity can translate into improved long-term outcomes without undue toxicity.

Patients with indolent lymphoma are treated episodically with chemotherapy, immunotherapy, or radiation often over a period of many years, sometimes decades. Therapy is initially highly effective in palliating symptoms and relieving potentially life-threatening complications, but it is not curative. Over time, response rates diminish and become less durable. The outcome for patients who are refractory to rituximab is particularly poor, and few alternative treatment options remain. In this context of heavily pretreated disease, the evidence supports the use of ^131^I–tositumomab. ^131^I–Tositumomab demonstrated significant anti-lymphoma activity in six single-arm trials in patients with nhl relapsed or refractory to chemotherapy without rituximab and in two single-arm phase ii trials in patients with nhl relapsed or refractory to rituximab with or without chemotherapy.

For most of this heavily pretreated patient population, therapeutic options have been exhausted. A standard comparison therefore does not exist. However, one trial used each patient as a paired self control for comparing duration of response with the patient’s last qualifying chemotherapy regimen 5. That trial reported a significant difference in objective response (65% with ^131^I–tositumomab vs. 28% with last chemotherapy), and 53% of patients had a longer response duration after ^131^I–tositumomab than after their most recent chemotherapy. This longer response may represent a beneficial effect on the natural history of the disease because, typically, a lower response rate and shorter duration of response are observed with each successive treatment. In addition, a proportion of responders had very long durable remissions. For patients with a cr, median duration of response was 47.2 months as compared with only 4.8 months for their last qualifying chemotherapy. Given the limited available treatment options for pretreated patients, the use of ^131^I–tositumomab may offer benefit when other treatments (including rituximab) have failed.

The role of ^131^I–tositumomab in individuals with transformed low-grade nhl is also of interest, given the poor prognosis associated with currently available therapies. An integrated analysis of patients with transformation across five trials documented moderate response rates and a median response duration of 20 months, results that were commensurate with the heavily pretreated low-grade population 43. However, this small and selected population of patients with an extended time from transformation until treatment with ^131^I–tositumomab may not be reflective of all patients with transformed nhl 45, and further prospective data for this unique presentation are warranted.

The data do not currently identify whether there is a differential benefit between the various indolent histologies (follicular vs. non-follicular) enrolled in these pivotal trials. The data supporting the use of ^131^I–tositumomab in previously untreated patients with nhl are limited. No randomized controlled trials have compared ^131^I–tositumomab with standard therapy, and therefore the use of ^131^I–tositumomab in this patient population should be reserved until evidence becomes available supporting improved clinical outcomes with ^131^I–tositumomab as compared with current standard therapies.

The evidence for ^131^I–tositumomab as part of a conditioning regimen before asct is limited to two single-arm trials 17,40. Although encouraging, the limited data preclude any clear conclusions of benefit in that setting.

The toxicities of ^131^I–tositumomab are predictable. The main toxicity is hematologic, with delayed-onset cytopenias whose nadirs occur at 7–9 weeks from treatment. Particular attention to severe myelosuppression is warranted for patients with known bone marrow involvement and thrombocytopenia preceding therapy. Dose reductions are required if platelets reach 100–150×10^9^/L, and the drug should not be administered if platelets are less than 100×10^9^/L, absolute neutrophil count is less than 1.5×10^9^/L, or bone marrow involvement is greater than 25%. The annualized incidence of mds and aml in patients with previously treated nhl is 1.4% per year and would be considered acceptable in this group of patients who have often received prior leukemogenic anti-lymphoma therapies such as alkylating agents. The incidence of hama varied from 0% to 35% and is of questionable clinical significance.

The evidence has highlighted a number of predictors for response to radioimmunotherapy. Common predictors for response include indolent-histology disease (compared with transformed histology), non-bulky disease, and fewer prior therapies. However, these results should be considered hypothesis-generating at this time, given the limited sample sizes on which the subgroup analyses were based.

The final question that guided this review was the role of dosimetry in establishing the safety and efficacy of ^131^I–tositumomab. Although dosimetric findings did not correlate with tumour response, dosimetry was performed in all clinical studies involving this agent and is currently mandated in North American and European jurisdictions to determine the patient-specific therapeutic dose. Although there may be logistic barriers to the performance of dosimetry, especially in smaller centres of practice, these limitations are out of the scope of the present review.

Currently, no comparative data addressing the use of one radioimmunoconjugate over another are available. Another radiolabelled anti-CD20 antibody, ^90^Y-ibritumomab tiuxetan (Zevalin: idec Pharmaceuticals, San Diego, CA, U.S.A.), is being studied predominantly in indolent and transformed lymphoma. Important differences in radiation characteristics and dosimetry requirements may limit the class generalizability of these antibodies.

The strengths of the present review include the use of validated methods for the performance of systematic reviews, extension of the literature search to include preliminary abstract data to minimize publication bias, and objective data abstraction according to predefined outcome questions. However, the review does have limitations. For most of the trials, we did not formally appraise study quality because they were phase ii studies and several were reported only in abstract form. This lack of appraisal limited the discrimination and utility of any methodologic grading scores. Also, the variability of the data precluded any pooling of results or use of meta-analytic summary techniques. We appreciate that the data come largely from single-arm studies and that the results are subject to selection bias; however, we have tempered our conclusions regarding this agent to reflect the currently available evidence. Finally, we acknowledge that the evidence regarding the role of ^131^I–tositumomab will continue to mature and evolve beyond this original systematic review and summary document. A current listing of phase iii trials is provided in Table IX, and we invite practitioners and patients to review the Web site of the pebc (www.cancercare.on.ca/cms/One.aspx?portalId=1377&pageId=10269) to remain abreast of the update process mandated for these guidelines.

Despite limitations, the data suggest a role for ^131^I–tositumomab in selected patients with nhl. The current evidence supports a role in the management of indolent lymphoma refractory to prior therapy that includes rituximab with or without chemotherapy. The precise role of ^131^I–tositumomab within the lymphoma armamentarium will undoubtedly continue to evolve.

## Figures and Tables

**TABLE I:** Literature search strategy

Step	Search term
1	zevalin.mp.
2	ibritumomab tiuxetan.mp.
3	anti-CD20.mp.^a^
4	antiCD20.mp.^a^
5	antiCD-20.mp.^a^
6	idec-y2b8.mp.
7	idecy2b8.mp.
8	idec-2b8.mp.
9	idec2b8.mp.
10	idec-In2b8.mp.
11	idecIn2b8.mp.
12	idec-129.mp.
13	idec129.mp.
14	or/1–13
15	lymphoma.mp.
16	exp lymphoma/
17	exp lymphoma, large-cell/b
18	or/15–17
19	14 and 18
20	limit 19 to human
21	limit 20 to English language
22	comment.pt.
23	letter.pt.
24	editorial.pt.
25	or/22–24
26	21 not 25^c^
27	20 not 21
28	27 not 25^d^

a Included in the original literature search (July 2003).

b Included in the May 2004 literature search.

c Results for citations in the English language.

**TABLE II:** Primary and additional publications of trials included in this systematic review

Primary publication	Publications with additional information
Press *et al.,* 1993^8^	Liu *et al.,* 1998^9^
	Gopal *et al.,* 2003^10^
Press *et al.,* 1995^11^	Liu *et al.,* 1998^9^
	Gopal *et al.,* 2003^10^
Kaminski *et al.,* 2000^12^	Kaminski *et al.,* 1993^13^
	Kaminski *et al.,* 1996^14^
	Wahl *et al.,* 1998^15^
	Bennet *et al.,* 2005^16^
Press *et al.,* 2000^17^	Gopal *et al.,* 2002^18^
Vose *et al.,* 2000^19^	Kaminski *et al.,* 2001^20^ (abstract)
	Sgouros *et al.,* 2003^21^
	Bennet *et al.,* 2005^16^
Kaminski *et al.,* 2001^5^	Kaminski *et al.,* 2001^20^ (abstract)
	Sgouros *et al.,* 2003^21^
	Bennet *et al.,* 2005^16^
Davis *et al.,* 2003^22^ (abstract)	Bennet *et al.,* 2005^16^
Kaminski *et al.,* 2003^23^ (abstract)	Kaminski *et al.,* 2005^24^,^a^
Press *et al.,* 2003^25^	Press *et al.,* 2006^26^,^a^
Zelenetz *et al.,* 2003^27^ (abstract)	None
Davies *et al.,* 2004^28^	None
Leonard *et al.,* 2004^29^ (abstract)	Leonard *et al.,* 2005^30^,^a^
Link *et al.,* 2004^31^ (abstract)	None
Mones *et al.,* 2004^32^ (abstract)	Mones *et al.,* 2007^33^,^a^
Horning *et al.,* 2005^34^	Bennet *et al.,* 2005^16^
Kaminski *et al.,* 2005^35^	Koral *et al.,* 2000^21^
	Koral *et al.,* 2000^36^
	Koral *et al.,* 2002^37^
	Koral *et al.,* 2003^38^
	Bennet *et al.,* 2005^16^
Nair *et al.,* 2005^39^ (abstract)	None
Vose *et al.,* 2005^40^	None

a See Appendix A for details regarding this publication.

**TABLE III:** Trials of ^131^I tositumomab (^131^itb) in patients with previously treated non-Hodgkin lymphoma (nhl): study characteristics

Reference	Study type	Patient characteristics	Intervention	Pts (*n*)^a^
*Relapsed or refractory to chemotherapy without rituximab*				
Press *et al.,* 1993^8^	Single-arm	CD20+ or CD37+ B-cell nhl unresponsive to conventional systemic therapy	^131^itb phase i [total body dose: 10–31 Gy (dose escalation)], autologous stem cell transplantation if needed	12^b^
Press *et al.,* 1995^11^	Single-arm	CD20+ nhl relapsed after at least one chemotherapy regimen	^131^itb (total body dose: 25–31 cGy), autologous stem cell transplantation or peripheral stem cell transplantation if needed	25
Kaminski *et al.,* 2000^12^	Single-arm	Relapsed or refractory CD20+ B-cell nhl	^131^itb phase i/ii (phase ii total body dose: 75 cGy)	59
Vose *et al.,* 2000^19^	Single-arm	Low-grade or transformed low-grade CD20+ nhl relapsed or refractory to at least one anthracycline- or anthracenedione-containing chemotherapy regimen	^131^itb (total body dose: 75 cGy, 65 cGy if platelets ≤ 149,000/mm^3^)	47
Kaminski, 2001^5^	Single-arm	Low-grade or transformed low-grade CD20+ B-cell nhl relapsed or refractory after at least two prior chemotherapy regimens	^131^itb (total body dose: 75 cGy, 65 cGy if platelets < 150,000/mm^3^)	60
Davis *et al.,* 2003^22^ (abstract)	*Randomized*	Relapsed or refractory CD20+ nhl	^131^itb (total body dose: nr) Unlabelled tositumomab	42 36
Davies *et al.,* 2004^28^	Single-arm	B-Cell nhl in first or second recurrence	^131^itb (total body dose: 75 cGy, 65 cGy if platelets ≤ 149,000/mm^3^)	44
*Relapsed or refractory to rituximab with or without chemotherapy*				
Horning *et al.,* 2005^34^	Single-arm	Indolent or transformed nhl relapsed or refractory to rituximab	^131^itb (total body dose: 75 cGy, 65 cGy if platelets < 150,000/mm^3^)	43
Nair *et al.,* 2005^39^ (abstract)	Single-arm	CD20+ nhl refractory to chemotherapy plus rituximab	^131^itb (total body dose: 75 cGy, 65 cGy if platelets < 150,000/mm^3^)	11
*^131^itbconditioning for autologous stem cell transplantation*				
Press *et al.,* 2000^17^	Single-arm	CD20+ nhl relapsed or refractory to previous chemotherapy, bone marrow involvement < 25%	^131^itb [total body dose: 20–27 Gy (dose escalation)], followed by etoposide (60 mg/kg), plus cyclophosphamide (100 mg/kg), plus autologous stem cell transplantation	52
Vose *et al.,* 2005^40^	Single-arm	Previously treated chemotherapy-resistant CD20+ aggressive nhl	^131^itb [total body dose: 30–75 cGy (dose escalation)], followed by beam (carmustine 300 mg/m^2^ day 1; plus etoposide 100 mg/m^2^ twice daily, days 2–5; plus cytarabine 100 mg/m^2^ twice daily, days 2–5; plus melphalan 140 mg/m^2^ day 6), plus autologous stem cell transplantation (day 7)	23
*^131^itbin alternative regimens*				
Kaminski *et al.,* 2003^23^ (abstract)	Single-arm	Low-grade or transformed low-grade nhl previously treated with ^131^itb	^131^itb phase i (total body dose: nr)	32
Mones *et al.,* 2004^32^ (abstract)	Single-arm	Relapsed or refractory low-grade nhl, bone marrow involvement > 25%	^131^itb phase i [total body dose: 45cGy (10 cGy dose-escalation increments)]	11

a Randomized or enrolled/eligible.

b Of the 43 enrolled patients, 19 received therapeutic doses, and only 12 of the 19 received ^131^itb.

Pts = patients; nr= not reported.

**TABLE IV:** Trials of ^131^I–tositumomab (^131^itb) in patients with previously treated non-Hodgkin lymphoma: response and survival

Reference	Study type	Intervention	Pts (n)	or (%)	cr (%)		Median results (months) for		
						ttp	Response duration	os	Follow-up
*Relapsed or refractory to chemotherapy without rituximab*									
Press *et al.,* 1993^8^	Single-arm	^131^itb phase i [total body dose: 10–31 Gy (dose escalation)]	12^a^	100^b^	83	nr	11	21+	26
Press *et al.,* 1995^11^	Single-arm	^131^itb (total body dose: 27 Gy)	21^c^	90^b^	76	Not yet reached	nr	Not yet reached	12
Kaminski *et al.,* 2000^12^	Single-arm	^131^itb phase i/ii (phase ii total body dose: 75 cGy)	59	71	34	12	nr	41^d^	37.2
Vose *et al.,* 2000^19^	Single-arm	^131^itb (total body dose: 75 cGy, 65 cGy if platelets ≤ 149,000/mm^3^)	47	57	32	11.6	9.9	36	nr
Kaminski *et al.,* 2001^5^	Single-arm	^131^itb (total body dose: 75 cGy, 65cGy if platelets < 150,000/mm^3^)	60	65	20	8.4	6.5	22.8	nr
Davis *et al.,* 2003^22^ (abstract)	*Randomized*	^131^itb (total body dose: nr)	42	55	33	6.3	Not yet reached	nr	42.6
		Unlabelled tositumomab	36	19	8	5.5	28.1	nr	
				*p*=0.002		*p*=0.031			
Davies *et al.,* 2004^28^	Single-arm	^131^itb (total body dose: 75 cGy, 65 cGy if platelets ≤ 149,000/mm^3^)	41^e^	76	49	9.6	15	Not yet reached	36
*Relapsed or refractory to rituximab with or without chemotherapy*									
Horning *et al.,* 2005^34^	Single-arm	^131^itb (total body dose: 75 cGy, 65 cGy if platelets < 150,000/mm^3^)	40^f^	65	38	10.4	nr	Not yet reached	39
Nair *et al.,* 2005^39^ (abstract)	Single-arm	^131^itb (total body dose: 75 cGy, 65 cGy if platelets < 150,000/mm^3^)	11	72	27	nr	nr	nr	26
^131^*itbconditioning for autologous stem cell transplantation*									
Press *et al.,* 2000^17^	Single-arm	^131^itb [total body dose: 20–27 cGy (dose escalation)], followed by etoposide, plus cyclophosphamide, plus autologous stem cell transplantation	52	87^g^	77^g^	40^h^	nr	2-Year: 83%	nr
Vose *et al.,* 2005^40^	Single-arm	^131^itb [total body dose: 30–75 cGy (dose escalation)], followed by beam (carmustine, etoposide, cytarabine, melphalan), plus autologous stem cell transplantation	23	65	57	34^i^	nr	34^d^	38
^131^*itbin alternative regimens*									
Kaminski *et al.,* 2003^23^ (abstract)	Single-arm	^131^itb phase i (total body dose: nr), previous treatment with ^131^itb	32	56	22	11.8	10.7	nr	26
Mones *et al.,* 2004^32^ (abstract)	Single-arm	^131^itb phase i [total body dose: 45 cGy (dose escalation)], bone marrow involvement > 25%	11	18	nr	nr	nr	nr	nr

a Only 12 of the 43 enrolled patients received a therapeutic dose of ^131^itb.

b Includes patients with complete, partial, or minor response.

c Of 25 enrolled patients, 4 did not receive treatment and were not included in the response and survival data analysis.

d Estimated from Kaplan–Meier survival curve.

e Of 44 enrolled patients, 3 did not receive treatment and were not included in the final analysis.

f Of 43 enrolled patients, 3 did not receive treatment and were not included in the final analysis.

g Response rates were calculated based on 31 patients that were evaluable for response.

h Estimated from Kaplan–Meier progression-free survival curve.

i Event-free survival, estimated from Kaplan–Meier event-free survival curve.

**TABLE V:** Trials of ^131^I tositumomab (^131^itb) in patients with previously treated non-Hodgkin lymphoma: adverse events

Reference	Study type	Intervention	Pts (n)	Thrombo- cytopenia (grades 3–4) (%)	Neutropenia (grades 3–4) (%)	Anemia (grades 3–4) (%)	Infection (grades 1–4) (%)	Febrile neutropenia (%)	Human anti-mouse antibody (%)
*Relapsed or refractory to chemotherapy without rituximab*									
Press *et al.,* 1993^8^	Single-arm	^131^itb phase i [total body dose: 10–31 Gy (dose escalation)]	43^a^	nr	nr	nr	21	nr	7
Press *et al.,* 1995^11^	Single-arm	^131^itb (total body dose: 27 Gy)	21^b^	nr	nr	nr	38^c^	nr	19
Kaminski *et al.,* 2000^12^	Single-arm	^131^itb phase i/ii (phase ii total body dose: 75 cGy)	59	40	55	10	22	nr	17
Vose *et al.,* 2000^19^	Single-arm	^131^itb (total body dose: 75 cGy, 65 cGy if platelets ≤ 149,000/mm^3^)	47	nr	11 (grade 4)	nr	24	nr	2
Kaminski *et al.,* 2001^5^	Single-arm	^131^itb (total body dose: 75 cGy, 65 cGy if platelets < 150,000/mm^3^)	60	2 (grade 4)	18 (grade 4)	0 (grade 4)	25	2	8
Davis *et al.,* 2003^22^ (abstract)	*Randomized*	^131^itb (total body dose: nr)	42	12 (grade 4)	17 (grade 4)	5 (grade 4)	nr	nr	27
		Unlabelled tositumomab	36	0 (grade 4)	3 (grade 4)	0 (grade 4)	nr	nr	19
Davies *et al.,* 2004^28^	Single-arm	^131^itb (total body dose: 75 cGy, 65 cGy if platelets ≤ 149,000/mm 3)	41^d^	32	45	5	15 (grade 3–4)	5	10
*Relapsed or refractory to rituximab with or without chemotherapy*									
Horning *et al.,* 2005^34^	Single-arm	^131^itb (total body dose: 75 cGy, 65 cGy if platelets < 150,000/mm^3^)	40^e^	25	42	10	55	nr	0
Nair *et al.,* 2005^39^ (abstract)	Single-arm	^131^itb (total body dose: 75 cGy, 65 cGy if platelets < 150,000/mm^3^)	11	18 (grade 4)	nr	nr	nr	nr	nr
*^131^itbconditioning for autologous stem cell transplantation*									
Press *et al.,* 2000^17^	Single-arm	^131^itb [total body dose: 20–27 cGy (dose escalation)], followed by etoposide, plus cyclophosphamide, plus autologous stem cell transplantation	52	100	100 (grade 4)	nr	71^f^	nr	13
Vose *et al.,* 2005^40^	Single-arm	^131^itb [total body dose: 30–75 cGy (dose escalation)], followed by beam (carmustine, etoposide, cytarabine, melphalan), plus autologous stem cell transplantation	23	100	100 (grade 4)	nr	52	>90%	35
*^131^itbin alternative regimens*									
Kaminski *et al.,* 2003^23^ (abstract)	Single-arm	^131^itb (total body dose: nr)	32	38	44	nr	50	3	10
Mones *et al.,* 2004^32^ (abstract)	Single-arm	^131^itb [total body dose: 45 cGy (10 cGy dose escalation increments)]	11	nr	nr	nr	nr	nr	nr

a Twelve patients received a therapeutic dose of ^131^itb; the number of patients that received a dosimetric dose of ^131^itb was not reported.

b Of 25 enrolled patients, 4 did not receive treatment and were not included in the final analysis.

c Two patients had grade 3 or 4 infection, and one patient died from infection (grade 5).

d Of 44 enrolled patients, 3 did not receive treatment and were not included in the final analysis.

e Of 43 enrolled patients, 3 did not receive treatment and were not included in the final analysis.

f Four patients had grade 3 or 4 infection.

Pts = patients; nr= not reported.

**TABLE VI:** Single-arm trials of ^131^I–tositumomab (^131^itb) in patients with previously untreated non-Hodgkin lymphoma (nhl): study characteristics

Reference	Patient characteristics	Intervention	Pts (*n*)^a^
Press *et al.,* 2003^25^	Previously untreated CD20+ stage ii–iv follicular nhl	chop (cyclophosphamide 750 mg/m^2^ day 1, plus doxorubicin 50 mg/m^2^ day 1, plus vincristine 1.4 mg/m^2^ day 1, plus prednisone 100 mg days 1–5) every 21 days for 6 cycles, followed by ^131^itb (total body dose: 75 cGy)	90
Zelenetz *et al.,* 2003^27^ (abstract)	Previously untreated mantle-cell lymphoma	^131^itb (total body dose: nr), followed 13–16 weeks later by chop^b^	13
Leonard *et al.,* 2004^29^ (abstract)	Previously untreated advanced low-grade nhl	Fludarabine 25 mg/m^2^ daily for 5 days, every 5 weeks for 3 cycles, followed by ^131^itb (total body dose: 75 cGy)	38
Link *et al.,* 2004^31^ (abstract)	Previously untreated follicular nhl	cvp (cyclophosphamide 400 mg/m^2^ days 1–5, plus vincristine 1.4 mg/m^2^ day 1, plus prednisone 100 mg/m^2^ days 1–5) every 21 days for 6 cycles, followed by ^131^itb (total body dose: 75 cGy)	30
Kaminski *et al.,* 2005^35^	Previously untreated advanced-stage follicular nhl	^131^itb (total body dose: 75 cGy)	76

a Number enrolled and eligible.

b Standard chop, but with a cyclophosphamide dose of 1000 mg/m^2^.

Pts = patients; nr= not reported.

**TABLE VII:** Single-arm trials of ^131^I–tositumomab (^131^itb) in patients with previously untreated non-Hodgkin lymphoma: response and survival

Reference	Intervention	Pts	or	cr		Median results (months) for		
		(*n*)^a^	(%)	(%)	ttp	Response duration	os	Follow-up
Press *et al.,* 2003^25^	chop followed by ^131^itb (total body dose: 75 cGy)	90	90	67	Not yet reached	nr	Not yet reached	27.6
Zelenetz, 2003^27^ (abstract)	^131^itb (total body dose: nr) followed by chop	12^b^	75	75	nr	Not yet reached	nr	11
Leonard *et al.,* 2004^29^ (abstract)	Fludarabine, followed by ^131^itb (total body dose: 75 cGy)	35^c^	100	83	Not yet reached	nr	nr	52.8
Link *et al.,* 2004^31^ (abstract)	cvp followed by ^131^itb (total body dose: 75 cGy)	30	100	80	Not yet reached	nr	nr	27.6
Kaminski *et al.,* 2005^35^	^131^itb (total body dose: 75 cGy)	76	95	75	73.2	nr	Not yet reached	61.2

a Number included in the analysis.

b Of 13 enrolled patients, 1 did not receive treatment with ^131^itb and was not included in the analysis of the data.

c Of 38 enrolled patients, 3 did not receive treatment with ^131^itb and were not included in the analysis of the data.

Pts = patients; or= complete response, unconfirmed complete response, and partial response; cr= complete response and unconfirmed complete response; ttp= time to progression; os= overall survival; chop= cyclophosphamide, doxorubicin, vincristine, prednisone; nr= not reported; cvp= cyclophosphamide, vincristine, and prednisone.

**TABLE VIII:** Single-arm trials of ^131^I–tositumomab (^131^itb) in patients with previously Untreated non-Hodgkin lymphoma: adverse events

Reference	Intervention	Pts (*n*)^a^	Thrombocytopenia (grades 3–4) (%)	Neutropenia (grades 3–4) (%)	Anemia (grades 3–4) (%)	Infection (grades 1–4) (%)	Febrile neutronpenia (%)	Human anti-mouse antibody (%)
Press *et al.,* 2003^25^	chop followed by ^131^itb (total body dose: 75 cGy)	82^b^	11	13	2	2 (grade 3)	nr	nr
Zelenetz *et al.,* 2003^27^ (abstract)	^131^itb (total body dose: nr) followed by chop^a^	12^c^	nr	nr	nr	nr	42	16
Leonard *et al.,* 2004^29^ (abstract)	Fludarabine followed by ^131^itb (total body dose: 75 cGy)	35^d^	29 (grade 4)	34 (grade 4)	3 (grade 4)	nr	nr	6
Link *et al.,* 2004^31^ (abstract)	cvp followed by ^131^itb (total body dose: 75 cGy)	30	23 (grade 4)	33 (grade 4)	nr	nr	nr	0
Kaminski *et al.,* 2005^35^	^131^itb (total body dose: 75 cGy)	76	17	34	0	nr	0	63

a Number included in analysis.

b Of 90 enrolled patients, 9 were not evaluable for toxicity.

c Of 13 enrolled patients, 1 did not receive treatment with ^131^itb and was not included in the analysis of the data.

d Of 38 enrolled patients, 3 did not receive treatment with ^131^itb and were not included in the analysis of the data.

Pts = patients; chop= cyclophosphamide, doxorubicin, vincristine, prednisone; nr= not reported; cvp= cyclophosphamide, vincristine, and prednisone.

**TABLE IX:** Ongoing comparative or phase iii trials

Protocol ID	Title and details of trial
swog S0016 NCT00006721 calgb 50102	Phase iii Randomized Study of Cyclophosphamide, Doxorubicin, Vincristine, and Prednisone (chop) with Either Rituximab or Iodine ^131^I Tositumomab (Monoclonal Antibody Anti-B1) in Patients with Newly Diagnosed Follicular Non-Hodgkin’s Lymphoma
	***Outcomes:*** Progression-free survival, overall survival, response, toxicity
	***Projected accrual:*** 500 patients
	***Status:*** Study is ongoing, but not recruiting participants
	***Notes:*** A chop-only arm closed to recruitment on December 15, 2002
	***Summary last modified:*** April 14, 2009
	***Accessed:*** May 10, 2009
	***Available at:***www.clinicaltrials.gov/ct2/show/NCT00006721
bmt CTN0401 NCT00329030	Phase iii Rituxan/beam vs. Bexxar/beam with Autologous Hematopoietic Stem Cell Transplantation (asct) for Persistent or Relapsed Chemotherapy Sensitive Diffuse Large B-Cell Non-Hodgkin’s Lymphoma
	***Outcomes:*** Progression-free survival, overall survival, response, toxicity
	***Projected accrual:*** 224 patients
	***Status:*** Currently recruiting
	***Summary last modified:*** April 8, 2009
	***Accessed:*** May 10, 2009
	***Available at:***www.clinicaltrials.gov/ct2/show/NCT00329030
CCBX001-053 NCT00078676 NCT00319332	A Comparative Study of Iodine I-131 Tositumomab Therapeutic Regimen Versus Ibritumomab Tiuxetan Therapeutic Regimen
	***Status:*** Withdrawn
	***Summary last modified:*** January 24, 2007.
	***Accessed:*** May 10, 2009
	***Available at:***www.clinicaltrials.gov/ct2/show/NCT00319332
CCBX001-049 NCT00078598 NCT00268983	A Study of Rituximab Versus Iodine I-131 Tositumomab Therapy for Patients with Non-Hodgkin’s Lymphoma
	***Outcomes:*** Response and safety
	***Projected accrual:*** 506 patients
	***Status:*** Terminated
	***Summary last modified:*** November 8, 2005
	***Accessed:*** May 10, 2009
	***Available at:***www.clinicaltrials.gov/ct2/show/NCT00078598

## Footnotes

a Also see Appendix A, which describes an update to the original search and the articles located as a result.

## APPENDIX A UPDATED LITERATURE SEARCH

To ensure that our systematic review and conclusions remained valid, we updated the medline literature search outlined in Table I to May 10, 2009, from 2005, with a focus on randomized controlled trials and single-arm noncomparative studies. The same inclusion criteria that defined the original search were again applied. The new literature search identified 165 citations. Seven full publications met the inclusion criteria. No randomized phase iii studies were identified for this time period. All of the reports were single-arm phase ii reports. One publication, an integrated analysis of five pivotal studies on ^131^I–tositumomab, had already been included in the systematic review 44. Another study provided a longer-term update of a published report identified in the review 26; three other reports represented the final publication of abstracts identified in the review 33,24,30. Thus, only two new single-arm studies were discovered 41,42 (see Table A-I). The data from the updated reports and the two novel studies do not affect the summary answers to the questions that guided the original systematic review.

**TABLE A-I:** Novel and updated trials of ^131^I–tositumomab (^131^itb) in patients with non-Hodgkin lymphoma

Reference	Study type	Intervention	Pts	or	cr		Median results (months) for		
			(*n*)^a^	(%)	(%)	ttp	Response duration	os	Follow-up
*Previously untreated patients*									
Leonard *et al.,* 2005^30^ (final report after abstract 29)	Single-arm	Fludarabine followed by ^131^itb (total body dose: 75 cGy)	35	100	86	Not yet reached	nr	nr	58
Press *et al.,* 2006^26^	Single-arm	chop followed by ^131^itb (total body dose: 75 cGy)	90	91	69	5-Year: 67%	nr	5-Year: 87%	5.1 Years
*^131^**itb**in alternative regimens (previously treated patients)*									
Kaminski *et al.,* 2005^24^ (final report after abstract^23^)	Single-arm	^131^itb phase i (total body dose: nr), previous treatment with ^131^itb	32	56	25	11.8	15.2	nr	35
Mones *et al.,* 2007^33^ (final report after abstract 32)	Single-arm	^131^itb phase i [total body dose: 45 cGy (dose escalation)], bone marrow involvement > 25%	11	18	nr	nr	nr	nr	nr
*Relapsed or refractory to chemotherapy (previously treated patients)*									
Buchegger *et al.,* 2006^41^	Single-arm	^131^itb (total body dose: 75 cGy, 65 cGy if platelets ≤149,000/mm^3^)	18	81^b^	50^b^	22.5	nr	Not yet reached	48
*^131^**itb**conditioning for autologous stem cell transplantation*									
Gopal *et al.,* 2006^42^	Single-arm	^131^itb (total body dose: 25–27 cGy^c^), plus autologous stem cell transplantation	24	67	54	3-Year: 59%	nr	3-Year: 51%	2.9 Years

a Number included in the analysis.

b Calculated based on 31 patients that were evaluable for response.

c Dose to the critical normal organ predicted to receive the highest radiation exposure based on biodistribution study.

Pts = patients; or= complete response, unconfirmed complete response, and partial response; cr= complete response and unconfirmed complete response; ttp= time to progression; os= overall response; nr= not reported; chop= cyclophosphamide, doxorubicin, vincristine, prednisone.
